# Designer tRNAs for efficient incorporation of non-canonical amino acids by the pyrrolysine system in mammalian cells

**DOI:** 10.1093/nar/gkx1156

**Published:** 2017-11-21

**Authors:** Robert Serfling, Christian Lorenz, Maja Etzel, Gerda Schicht, Thore Böttke, Mario Mörl, Irene Coin

**Affiliations:** Institute of Biochemistry, Faculty of Life Sciences, University of Leipzig, Brüderstraße 34, 04103 Leipzig, Germany

## Abstract

The pyrrolysyl-tRNA synthetase/tRNA^Pyl^ pair is the most versatile and widespread system for the incorporation of non-canonical amino acids (ncAAs) into proteins in mammalian cells. However, low yields of ncAA incorporation severely limit its applicability to relevant biological targets. Here, we generate two tRNA^Pyl^ variants that significantly boost the performance of the pyrrolysine system. Compared to the original tRNA^Pyl^, the engineered tRNAs feature a canonical hinge between D- and T-loop, show higher intracellular concentrations and bear partially distinct post-transcriptional modifications. Using the new tRNAs, we demonstrate efficient ncAA incorporation into a G-protein coupled receptor (GPCR) and simultaneous ncAA incorporation at two GPCR sites. Moreover, by incorporating last-generation ncAAs for bioorthogonal chemistry, we achieve GPCR labeling with small organic fluorophores on the live cell and visualize stimulus-induced GPCR internalization. Such a robust system for incorporation of single or multiple ncAAs will facilitate the application of a wide pool of chemical tools for structural and functional studies of challenging biological targets in live mammalian cells.

## INTRODUCTION

Genetic code expansion is the most powerful method to introduce artificial moieties into proteins site-specifically in live cells. Non-canonical amino acids (ncAAs) are incorporated in response to an in-frame amber stop codon (UAG) by a suppressor tRNA (tRNA_CUA_), which is charged with the ncAA by an engineered amino-acyl-tRNA-synthetase (AARS). AARS/tRNA pairs evolved for ncAA mutagenesis in mammalian cells are mostly derived from the archaeal pair that naturally incorporates pyrrolysine (Pyl) in *Methanosarcina species* (PylRS/tRNA^Pyl^). Over a hundred ncAAs have been encoded using the Pyl system, including all ncAAs for ultrafast bioorthogonal chemistry (1), photo-caged and photo-switchable ncAAs, small fluorophores and photo-crosslinkers, among others (reviewed in (2)). However, the Pyl system gives modest protein yields in mammalian cells, which hampers its general applicability.

While non-canonical AARS/tRNA pairs must not cross talk with the translational pairs of the host cell (criterion of orthogonality), the tRNA must be compatible with the endogenous transcription and processing machinery. In mammalian cells, prokaryotic tRNAs that lack A-box and B-box internal promoter sequences (3), as in the case of tRNA^Pyl^, are transcribed by placing the tRNA gene under control of RNA Pol III external promoters, such as the H1 (4) or U6 promoter (5). Alternatively, some prokaryotic sequences have been engineered so as to have functional A and B-boxes (6). In the tRNA expression cassette, the gene lacks the universal CCA-end and is flanked by a T-rich 3′-trailer. In the cell, the trailer is enzymatically cleaved from primary transcripts and the 3′-CCA is added by the CCA-adding enzyme (7,8). In addition, multiple tRNA nucleotides are modified at specific positions, which includes isomerization of uridine to pseudouridine (U→Ψ), dehydrations and methylations (9). Once the mature tRNA is acylated by the cognate AARS, the AA-tRNA is bound by the elongation factor, which protects it from hydrolysis and delivers it to the ribosome, where it participates in protein synthesis (10).

**Figure 1.**
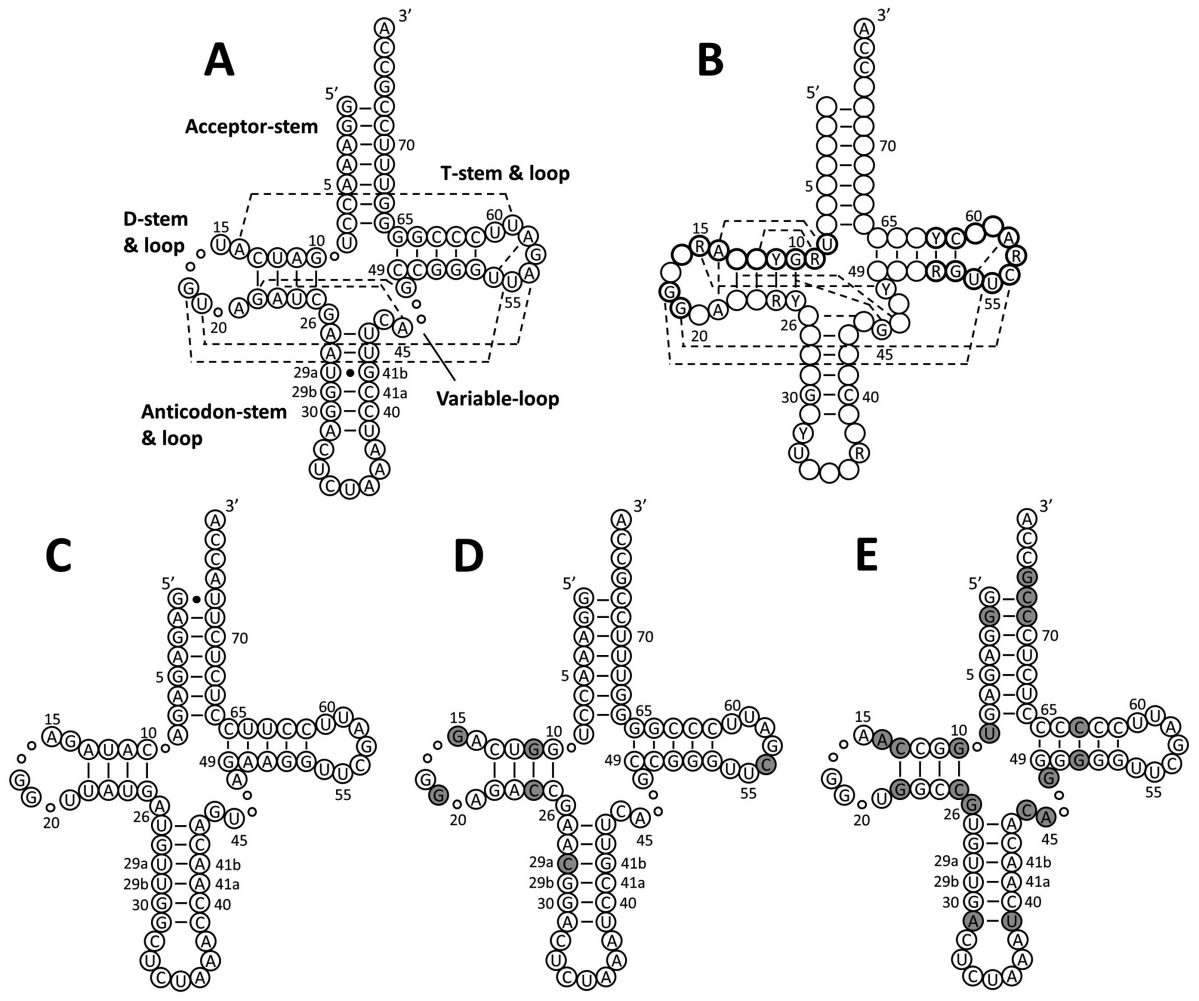
Cloverleaf structures of tRNAs. (**A**) *M. mazei* tRNA^Pyl^. Compared to canonical tRNAs, tRNA^Pyl^ misses nucleotides represented by small circles and features a longer anticodon stem (six base pairs instead of five). To conserve the canonical numbering (28,29), position 29:41 is counted twice as a/b. Dashed lines indicate tertiary interactions inferred from the crystal structure of *D. hafniense* tRNA^Pyl^ (11). (**B**) Canonical human tRNA with tertiary interactions indicated as dashed lines (26–28). Positions that are 100% conserved as a purine (R) or a pyrimidine (Y), and single nucleotides (A,U,G,C) that are >90% conserved are indicated. Nucleotides corresponding to the consensus internal promoter sequences (A-box, TRGCNNAGY for positions 8–16 and G18G19; B-box, GGTTCGANTCC for positions 52–62) are highlighted by bold circles. (**C**) Stabilized *B. taurus* mt-tRNA^Ser^_CUA_ amber suppressor (29). (**D**) tRNA^M15^. Gray shades indicate mutations from wild type tRNA^Pyl^. (**E**) tRNA^C15^. Gray shading indicates nucleotides imported from tRNA^Pyl^.

The archaeal tRNA^Pyl^ features a secondary structure that significantly diverges from that of canonical tRNAs (11) (Figure 1) and is shared in mammals only by the mitochondrial tRNA^Ser^_UGA_ (12). We suspected that such peculiar tRNA may be poorly compatible with the endogenous translational machinery and may possibly be unstable in the mammalian cytosol. In fact, it has been shown that stabilizing the U29a:G41b pair in tRNA^Pyl^ by the U29aC mutation (tRNA^Pyl^*) slightly improves ncAA incorporation rates in 293T cells (13). Experiments in *Escherichia coli* showed that the suppression efficiency by tRNA^Pyl^ is enhanced not only by the U29aC mutation (14), but also by improving the recognition with the endogenous elongation factor EF-Tu (15), as observed for other suppressor tRNAs (16).

In this work, we aimed at generating more efficient tRNA^Pyl^ variants for ncAA incorporation in mammalian cells. Since directed tRNA evolution using large libraries is not feasible in mammalian systems, we focused on screening small sets of rationally designed tRNAs. We selected two novel tRNAs that improve ncAA incorporation by several folds, thus enabling efficient ncAA incorporation into a G-protein coupled receptor (GPCR) and simultaneous ncAA incorporation at two GPCR sites. Moreover, by combining our novel tRNAs with a recently reported PylRS variant, we present the most efficient pyrrolysine system currently available for general application in mammalian cells.

## MATERIALS AND METHODS

### General molecular biology

*Escherichia coli* strain DH5α was used for cloning. PCRs were performed using Phusion High Fidelity DNA-Polymerase. Point mutations were introduced using the QuikChange^®^ method (Stratagene, La Jolla, CA, USA) adapted for Phusion polymerase. Oligonucleotides were purchased from Microsynth (Balgach, CH) and Biomers (Ulm, DE). All constructs were verified by DNA sequencing (Sequencing Laboratories Göttingen). Lys(Boc) and Lys(Z) were purchased from Bachem and Novabiochem (Merk), respectively, the other ncAAs from SiChem (Bremen, DE). ncAA stocks were prepared in NaOH at 0.1–0.5 M as described previously (17) right before the experiments.

All tRNA and PylRS expression cassettes were cloned into a modified pcDNA3.1 vector named pNEU (Supplementary Figure S1A). *Mb*PylRS^F^ and the monomeric U6-tRNA^Pyl^* cassette (Supplementary Figure S1A) were amplified from pAcBac1.tR4-MbPyl (P.G. Schultz, Addgene Plasmid #50832). The codon-optimized gene of *Mb*PylRS^F^ was custom synthesized by GeneArt (ThermoFisher Scientific). The NES motif was build *de novo* using overlapping primers and fused to the *Mb*PylRS via PCR. tRNA variants were mostly generated *de novo* using overlapping primers and joined to the U6 promoter via overlapping PCR. Tandem cassettes were generated as described previously (18). DNA sequences are reported in the supplementary data. The reporter plasmid for the fluorescence assay (Supplementary Figure S1B) was described previously (17). The pIRE4-Azi plasmid (Supplementary Figure S1C) bears a humanized gene encoding the E2AziRS (19) (synthesized by GeneArt) under control of the PGK promoter and four tandem repeats of the expression cassette for *Bacillus stearothermophilus* tyrosyl amber suppressor tRNA (*Bst*Yam) under control of the U6 promoter. The pcDNA3.1-CRF1R-FLAG and pcDNA3.1-CRF1R(xxxTAG)-FLAG plasmids were previously built by I.C. at The Salk Institute for Biological Studies (La Jolla, CA, USA) in the laboratory of Lei Wang (18).

### Cell culture

HEK293 and 293T cells were maintained at 37°C in a 5% CO_2_ atmosphere in Dulbecco's Modified Eagle's Medium (DMEM; high glucose, 4 mM glutamine, pyruvate), supplemented with 10% (v/v) of fetal bovine serum (FBS superior; Biochrom), 100 units/ml of Penicillin and 100 μg/ml Streptomycin. Unless otherwise stated, ncAAs were added to the growth medium 1 h prior transfection at a final concentration of 500 μM.

### Fluorescence assay

As previously described (17), ∼5 × 10^5^ HEK293 cells were seeded per well of 6-well plates in 2 ml complete growth medium and transfected the day after with 1 μg of the reporter plasmid and 1 μg of the tRNA/AARS plasmid using Polyethylenimine 25kD (PEI; Polysciences). Two days later, total green and red fluorescence of cell lysates was quantified using a plate reader (FLUOstar Omega, equipped with Ex485-12/Em520 and Ex584/Em620-10 filter sets). Suppression efficiency was calculated as the ratio between the fluorescence of the sample and the fluorescence obtained for wild type EGFP expression. All values were normalized to mCherry fluorescence.

### Isolation of total RNA and total small RNA

1.5 × 10^6^ HEK293 cells were seeded in 6 cm dishes and transfected the day after with 3 μg DNA plasmid encoding for the tRNA/AARS pair using PEI. Two days later, total RNA was isolated under acidic conditions using TRIzol^®^ (Invitrogen) according to the manufacturer protocol. RNA pellets were dissolved in 10 mM sodium acetate (pH 5.0). Large RNAs were precipitated with 10 M lithium chloride in 1:1 ratio (v/v) and separated by centrifugation at 14 000 × *g* and 4°C for 10 min. Small RNAs (<200 nt) were precipitated with ethanol and dissolved in RNase-free water.

### Semi-denaturing urea-PAGE and Northern blotting

Northern blot analysis was performed as described by Köhrer *et al.* (20) with minor variations for mini-gel format. Total tRNA samples (10 μg) were added of 2× sample buffer (0.1 M sodium acetate pH 5.0, 8 M urea, 0.05% bromophenol blue and 0.05% xylene cyanol), heated to 65°C for 5 min and loaded on freshly prepared semi-denaturing polyacrylamide gels (19:1 acrylamide/bisacrylamide) containing 7 M urea and 0.1 M sodium acetate pH 5.0. The gel was run at 5 W (100 V and 50 mA) in 0.1 M sodium acetate pH 5.0 for 3–4 h until the xylene cyanol reached the front. The RNA was transferred onto BrightStar™-Plus Nylon Membrane (ThermoFisher Scientific) at 2 mA/cm² in a semi-dry apparatus (Bio-Rad) for 35 min using transfer buffer (40 mM Tris–HCl, pH 8.0, 2 mM Na_2_EDTA). The membrane was irradiated at 254 nm UV light for 50 s (120 mJ/cm² total) and pre-hybridized for 14–20 h at 42°C in 6× saline–sodium citrate buffer (SSC) containing 10× Denhardt's solution and 0.5% SDS. 5′-biotin-labeled DNA oligonucleotides complementary to tRNA^Pyl^* and tRNA^M15^ (CGGAAACCCCGGGAATCxAACCCGGCTGAACGGA, x = deoxyribose spacer), tRNA^C15^ (CGGGAGAGGGGGGAATCGAACCCCCCTGTGTTGA), and ribosomal 5.8S RNA (CGCAAGTGCGTTCGAAGTGTCGATGATCAATGTG) were used as probes (100 pM) in the presence of a 100x excess (10 nM) of unlabeled ‘unfolder’-probes (tRNA^Pyl^ TCCGTTCGATCTACATGATCAGGTTTCC; tRNA^M15^ TCCGTTCGGTCTCCCTGACCAGGTTTCC; tRNA^C15^ TCAACACGGCCACCTTGGCCACTCTCCC). Membranes were hybridized for 12–24 h at 42°C in 6× SSC containing 0.1% SDS, washed at room temperature with 6× SSC (2 × 10 min), followed by washes at 4× and 2× SSC and incubated with streptavidin–horseradish peroxidase conjugate (1:10 000 in TBS + 0.1% Tween) for 1 h at room temperature. Membranes were washed with TBS + 0.1% Tween (3 × 10 min) and soaked in ECL reagent (0.1 M Tris–HCl pH 8.6, 22 % luminol, 10% *p*-coumaric acid, 10 % DMSO, 0.0001 % H_2_O_2_). After 1 min delay, signals were collected for 5 min in the dark (Gbox, Syngene). Signals were quantified using Image Studio software (LI-COR Biosciences, Ver5.2).

### Denaturing urea–PAGE

10 μg of total tRNA were added of 3x denaturing sample buffer (10 mM Tris–HCl, pH 7.6, 80% formamide (v/v), 0.25% bromophenol blue and 0.25% xylene cyanol) and incubated for 5 min at 65°C. Samples were loaded on fresh denaturing polyacrylamide gels containing 8 M urea and 1× TBE (89 mM Tris, 89 mM boric acid, 2 mM EDTA, pH 8.0). Electrophoresis was run at 100 V for 3 h in 1× TBE.

### *In vitro* tRNA transcription

tRNAs were transcribed from DNA constructs including 3′ and 5′ self-cleaving ribozymes (21) (Supplementary Figure S1D) using homemade T7 RNA polymerase (3 h, 37°C) followed by ribozyme cycling. Products were resolved on a denaturing urea-polyacrylamide gel (10%), tRNA bands were visualized via UV-shadowing and excised. tRNAs were extracted overnight in RNase-free water at 4°C, precipitated with ethanol and 3΄-dephosphorylated using T4 Polynucleotide Kinase (NEB) at 37°C for 4 h in 3′-dephosphorylation buffer (100 mM Imidazole, 10 mM MgCl_2_, 10 mM β-mercaptoethanol, 20 μg/ml BSA, 100 μM ATP, pH 6.0).

### In-line probing of tRNA secondary structure

*In vitro* tRNA was labeled at the 5′-end with [γ-^32^P]-ATP (Hartmann Analytic) using T4 Polynucleotide Kinase (New England Biolabs). The reaction mixture was resolved on a 10% denaturing urea-polyacrylamide gel, the labeled tRNA was eluted from the corresponding band overnight at 4°C in DEPC-treated water, precipitated with ethanol, and recovered in RNase-free water. In-line probing was performed as described (22). Labeled tRNA (∼10^5^ cpm) was denatured at 65°C for 5 min, refolded at RT for 5 min, and incubated in in-line reaction buffer (50 mM Tris–HCl, pH 8.5, 20 mM MgCl_2_ and 0.1 M KCl) for 40 h at 25°C in a 10 μl volume. In parallel, a ‘no-reaction’ control sample was incubated in RNase-free water. Reactions were quenched by adding 2× colorless loading dye (10 M urea and 1.5 mM EDTA). A third tRNA sample was digested in a reaction volume of 10 μl with 250 munits RNase T1 in RNase T1 buffer (10 mM Tris–HCl, pH 7.5, 30 mM NaCl and 0.5 mM EDTA) added of 2 μg *E. coli* total tRNA for 2–4 min at 37°C. The reaction was stopped on ice before adding colorless loading dye. Partial alkaline digestion of a fourth tRNA sample in NaHCO_3_ (100 mM, pH 9.75, 90°C, 2 min) provided the hydrolysis ladder. Samples were immediately resolved on a 10% denaturing polyacrylamide gel. Signals were detected with storage phosphor screens and analyzed with a Typhoon 9410 Phosphor imager (GE Healthcare).

### Detection of post-transcriptional modifications via reverse transcription (RT) based methods

Reverse transcription (RT) methods were applied to detect the presence of pseudouridine (Ψ), dihydrouridine (D) and 2′-*O*-methylations according to Motorin *et al.* (23) and Wintermeyer *et al.* (24). To detect pseudouridine (23) (Supplementary Figure S6), two 4 μg aliquots of the same total small tRNA sample were differently treated. One aliquot (sample Ψ) was added to 30 μl of 1-cyclohexyl-(2-morpholinoethyl)carbodiimide metho-*p*-toluene sulfonate (CMCT) solution (50 mM Bicine, pH 8.0, 7 M urea, 4 mM EDTA and 330 mM CMCT) and incubated at 37°C for 20 min. The RNA was precipitated with ethanol, dissolved in 50 mM Na_2_CO_3_ (aq) and incubated at 37°C for 3 h. The second aliquot (control C1) was treated exactly as sample Ψ, but omitting CMCT in the first buffer. The RNA was precipitated with ethanol and resuspended in nuclease-free water. 10 pmol of pre-treated *in vitro* transcribed tRNA or 1 μg total small tRNA in a volume of 10 μl were mixed with 20 pmol 5′-^32^P-labeled RT-primer (TGGCGGAAACCCCG for both tRNA^Pyl^* and tRNA^M15^, TGGCGGGAGAGGGG for tRNA^C15^), incubated at 65°C for 5 min and cooled on ice. Five units Avian Myeloblastosis Virus (AMV) Reverse Transcriptase (New England Biolabs) in AMV reaction buffer containing dNTPs to 1 mM were added. Reverse transcription was performed for 30 min at 42°C. Products were precipitated with ethanol, resuspended in 10 μl 2× colorless loading dye and resolved on 15% denaturing polyacrylamide gels. Sequencing ladders were generated via RT reactions, each containing an additional ddNTP in a ratio of 20:1 (ddNTP:dNTP). Radioactive signals were detected with storage phosphor screens and analyzed with a Typhoon 9410 Phosphor imager (GE Healthcare).

### Western blot analysis of CRF1 GPCR variants

The day before transfection, 4.5 × 10^5^ 293T cells were seeded on wells of six-well culture plates in 2 ml complete medium. For amber suppression, cells were transfected with equal amounts of the plasmid encoding for the orthogonal pair and pcDNA3.1-CRF1R(xxxTAG)-FLAG for a total of 2 μg DNA. For simultaneous suppression of amber and opal codons, cells were transfected with equal amounts of the two plasmids encoding for the orthogonal pairs (*Mb*PylRS^AF^/tRNA^Pyl^_UCA_ in pNEU, *Ec*AziRS/tRNA^Tyr^_CUA_ in pIRE4) and a third plasmid encoding for the CRF1R(xxxTGA, yyyTAG)-FLAG mutant in pcDNA3.1, for a total of 2.1 μg DNA. PEI was used as transfection reagent (3 μg PEI/1 μg DNA). Two days after transfection, whole cells lysates (15–50 μg) were resolved by SDS-PAGE, transferred to PVDF membranes and analyzed via Western blot using both an αFLAG and αACTB antibody, followed by ECL detection as described above.

### Fluorescence microscopy

1.0 × 10^5^ 293T cells were seeded per well of four-well IBIDI slides coated with poly-D-lysine hydrobromide (BD bioscience) in 600 μl FluoroBrite^®^ (Gibco) growth medium, supplemented with 10% (v/v) FBS, 100 units/ml Penicillin and 100 μg/ml Streptomycin. The following day, cells were co-transfected with the pCRF1R(xxxTAG)-EGFP plasmid and the plasmid encoding for the tRNA/AARS pair (1:1 w/w) for total of 0.4 μg DNA per well. 14–20 h later, 1.5 μM Tetrazine-Cy3 was added directly to the well for 5 min at 37°C (25). Cells were washed with growth medium (2 × 5 min). Genomic DNA was stained using Hoechst 33342. Fluorescence was detected on inverted fluorescence microscopes (Axio observer; Zeiss) equipped with following filters: Ex: BP470/40 Em: BP525/50 or Em: LP515 for green fluorescence (EGFP), Ex: BP565/30 Em: BP620/60 for red fluorescence (Cy3) and Ex: G365 Em: BP445/50 for blue/cyan fluorescence (Hoechst 33342).

## RESULTS

### Screening of rationally designed tRNA^Pyl^ variants

We designed two sets of tRNA^Pyl^ variants. The first set (M series) was based on the scaffold of *Methanosarcina mazei* tRNA^Pyl^ (Supplementary Table S1) and was aimed at improving the recognition of tRNA^Pyl^ by mammalian endogenous components. It included 11 tRNA^Pyl^ mutants in which single bases or base pairs were substituted with the corresponding nucleotides found in the conservation pattern of human tRNAs (26–28) (Figure 1B). In the D-stem, base pair U11:A24 was either inverted to A11:U24 or mutated to G:C to fit the human purine-pyrimidine conservation pattern. In the D-loop, the pyrimidine U15 was mutated to the conserved purine position (A/G). In the anticodon stem, C32 was substituted with the alternative pyrimidine U32. In the variable loop, G48 was mutated to A. In the T-loop, A56 was mutated to the conserved human counterpart C, and G57 to the alternative purine A. As A56 is engaged in a tertiary Watson-Crick pair by U19 in the D-loop (Figure 1A), we explored also the mutation U19G, both combined to A56C and on its own (Supplementary Table S1).

The second set (C series) relied on the structural similarity between the tRNA^Pyl^ and the mt-tRNA^Ser^_UGA_. In *E. coli*, chimera tRNAs built by introducing identity elements for PylRS into a stabilized CUA anticodon variant of bovine mt-tRNA^Ser^_UGA_ (Figure 1C) are acylated by the PylRS and function as orthogonal amber suppressors (29). We expected mt-tRNA^Ser/Pyl^ chimeras to be orthogonal in mammalian cells, as the cytosolic translational machinery is quite distinct from the mitochondrial one (30,31). On the other hand, we envisioned that the scaffold of a mammalian mitochondrial tRNA may provide tRNAs compatible with the mammalian context. Based on the known PylRS–tRNA^Pyl^ interaction pattern (11), we transplanted different combinations of recognition motifs for PylRS into *Bos taurus* mt-tRNA^Ser^_CUA_. These included the discriminator base G73, pairs G1:C72 and G2:C71 in the acceptor stem, U8 at the junction between acceptor stem and D-stem, pairs G10:C25 and C13:G22 in the D-stem, pair A31:U39 in the anticodon stem, C44, A45 and G48 in the variable loop, pairs C50:G64 and G51:C63 in the T-stem. We generated a set of 12 chimeric tRNAs, which included the two tRNA^Ser/Pyl^ chimeras described in the literature (29) (Supplementary Table S1).

**Figure 2.**
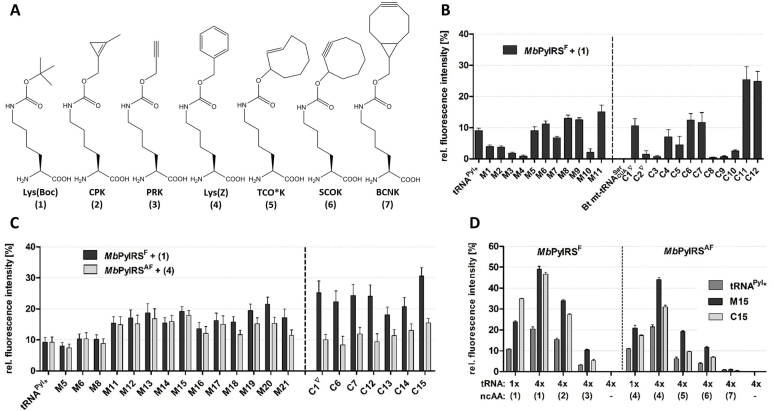
Selection of enhanced tRNA^Pyl^ variants for ncAA incorporation. (**A**) ncAAs used in this study. (B–D) Amber codon suppression in EGFP^Y183TAG^ by tRNA^Pyl^* and engineered tRNA variants. Bars represent the fluorescence of the probe relative to the fluorescence intensity of wild type EGFP expression. All values were normalized to mCherry fluorescence. Data represent the average ± SD of biological triplicates. All tRNA sequences are reported in Supplementary Table S1. (**B**) First round of screening. Mutant and chimera tRNAs (1 copy) were combined with *Mb*PylRS^F^ (natural gene with archaeal codon usage) for the incorporation of Lys(Boc). (**C**) Second round of screening. tRNA variants (1 copy) were combined with either *Mb*PylRS^F^ or *Mb*PylRS^AF^ (genes optimized for human codon usage) for incorporation of either Lys(Boc) or Lys(Z). (**D**) Incorporation efficiency of different ncAAs by tRNA^Pyl^*, tRNA^M15^, tRNA^C15^, using constructs bearing either one (1×) or four (4×) copies of the tRNA expression cassette.

We built bicistronic plasmids combining each tRNA with the PylRS from *Methanosarcina barkeri*, which bears the mutation Y349F for enhanced activity (PylRS^F^), as described for the homologous PylRS from *M. mazei* (32) (Supplementary Figure S1). Efficiency and orthogonality of the tRNAs were tested in HEK293 cells using the amber suppressor reporter EGFP^Y183TAG^ in a fluorescence assay (17). All tRNAs were orthogonal and gave no amber suppression in the absence of ncAA (Supplementary Table S2). In the presence of the PylRS substrate Lys(Boc) (Figure 2A), four tRNAs in the M series and five in the C series yielded higher amber suppression compared to the tRNA^Pyl^*, whereas the other tRNAs showed either lower efficiency or no amber suppression at all (Figure 2B). In the M series, the best tRNA was obtained by mutating the tertiary Watson-Crick pair U19:A56 between D-loop and T-loop to G19:C56. Further beneficial mutations were found in the D-stem (A11:U24 to G:C or C:G) and D-loop (U15G). However, the most efficient tRNAs were found in the C-series, with Chimera 11 and Chimera 12 yielding a 2.5-fold increase in suppression efficiency compared to tRNA^Pyl^*. In general, chimeras bearing the highest number of bases from tRNA^Pyl^ were the most efficient. Interestingly, the most efficient tRNAs of both series, Mutant 11 and the two chimeras, bear a canonical B-box in the T-arm.

We also considered the scaffold of *Desulfitobacterium hafniense* tRNA^Pyl^ (11,33). However, neither wild type *Dh*tRNA^Pyl^, nor a GC stabilized variant of it (Supplementary Table S1) performed better than the *Mm*tRNA^Pyl^* (Supplementary Figure S2).

We then discarded tRNAs less efficient than tRNA^Pyl^* and generated further tRNAs for a second round of screening (Supplementary Table S1). In the M series, we combined beneficial mutations found in the first round (Mutants 12–15). In addition, we attempted to reconstruct a complete A-box motif in the D-arm (Mutants 16–17). Finally, we explored different combinations of bases in the region connecting the acceptor stem to the T-arm (mutants 18–21), as mutations in this region have been shown to improve interactions with EF-Tu in bacteria (15). In the C series, we also reconstructed stepwise the A-box motif in the D-Arm (chimeras 13–15). As mutations in the tRNA may differently affect incorporation of different ncAAs (15,34), we tested now the incorporation of two sterically distinct ncAAs, Lys(Boc) and Lys(Z) (Figure 2A). Lys(Z) is recognized by the PylRS^AF^, which bears the F271A mutation deep inside the AA binding pocket to accept bulky substrates (32). We observed different trends with the two ncAAs in the two tRNA series. In general, with exception of the tRNA variants mutated in the putative region of elongation factor recognition (M18-M21), mutant tRNAs based on tRNA^Pyl^ (M series) showed parallel improvements with the two ncAAs, whereas all chimeras performed better with Lys(Boc) (Figure 2C).

For further experiments, tRNA^M15^ was selected from the M series because it gave the best overall performance considering both Lys(Boc) and Lys(Z), whereas tRNA^C15^ was selected from the C series, as the best tRNA for Lys(Boc) incorporation. To maximize suppression yields, we combined four tandem repeats of the tRNA expression cassette (Figure 2D). We then tested the two tRNAs for incorporation of several ncAAs by either PylRS^F^ or PylRS^AF^ (35–38). Compared to tRNA^Pyl^*, both tRNA^M15^ and tRNA^C15^ gave higher amber suppression yields with all ncAAs, with tRNA^M15^ outperforming tRNA^C15^ (Figure 2D).

### Characterization of the engineered tRNAs

**Figure 3.**
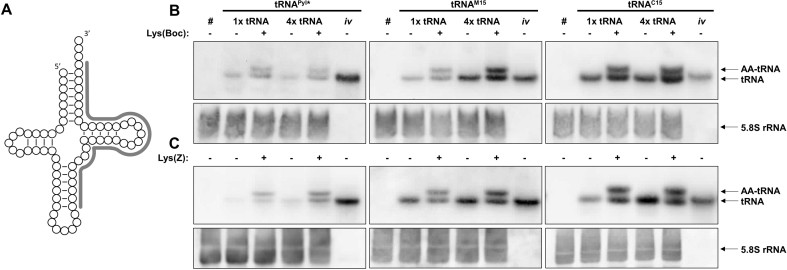
Northern blots. Total RNA from HEK293 cells expressing the indicated tRNA were isolated under acidic conditions to preserve the AA-tRNA ester linkage (39), resolved on 12.5% acidic urea-PA gels and electroblotted. (**A**) tRNA^Pyl^* and tRNA^M15^ were detected using the same 5′-biotinylated oligonucleotide probe (in gray), whereas tRNA^C15^ was detected with a different oligonucleotide targeted to the same tRNA region. (B and C) # indicates RNA probes extracted from non-transfected cells; *iv* indicates *in vitro* transcripts (loading controls, 25 ng); (**B**) cells co-expressed *Mb*PylRS^F^, (**C**) cells co-expressed *Mb*PylRS^AF^. The results are representative of at least 3 independent experiments.

The intracellular concentration and the amino acylation rate of tRNA^Pyl^*, tRNA^M15^ and tRNA^C15^ was examined via Northern Blot analysis (39) (Figure 3), using either one or four tRNA expression cassettes, in the presence and absence of either Lys(Boc) or Lys(Z). Compared to the loading controls, tRNA^C15^ showed the highest intracellular concentration, followed by tRNA^M15^ and tRNA^Pyl^* (Figure 3 and Supplementary Figure S3). In general, we detected more tRNA when the ncAA was present. The overall amounts of all tRNAs increased when increasing the number of tRNA expression cassettes. tRNA^C15^ showed the smallest increase, possibly explaining why this tRNA is the most efficient tRNA for Lys(Boc) incorporation when using one copy of the tRNA expression cassette but is equivalent to tRNA^M15^ when using four copies (Figure 2D). The acylation rate did not correlate either with the tRNA amount nor with the ncAA identity and was around 50% for all tRNAs, both with Lys(Boc) and Lys(Z) (Supplementary Table S3).

Notably, although all tRNAs run at the same height in fully denaturing gels, both *in vivo* and *in vitro* transcripts of tRNA^M15^ and tRNA^C15^ tRNAs run slightly faster than tRNA^Pyl^* in semi-denaturing gels (Supplementary Figure S4), which suggests that they remain partially folded. No major differences were identified by analyzing the secondary loops/stems structure of the three tRNAs with in-line probing (22,40) (Supplementary Figure S5), so that the higher stability of tRNA^M15^ and tRNA^C15^ is likely explained by more favorable tertiary interactions.

Using reverse transcription (RT)-based methods, we also investigated the occurrence of post-transcriptional modifications. While we did not find evidences for the presence of dihydrouridine and 2′-*O*-methylations, our RT results suggest the presence of the almost universally conserved Ψ55 in the T-loop of both tRNA^Pyl^* and tRNA^M15^, as well as the additional presence of Ψ39 in tRNA^M15^ (Supplementary Figure S6). Ψ39 is conserved in canonical tRNAs, where it stabilizes interactions at the base of the anticodon stem by providing an additional locus for hydrogen bonding to A31 (41,42). On the contrary, RT-methods did not provide clear indication for the presence of pseudouridine in tRNA^C15^.

### Testing tRNA^M15^ for ncAA incorporation into CRF1R

**Figure 4.**
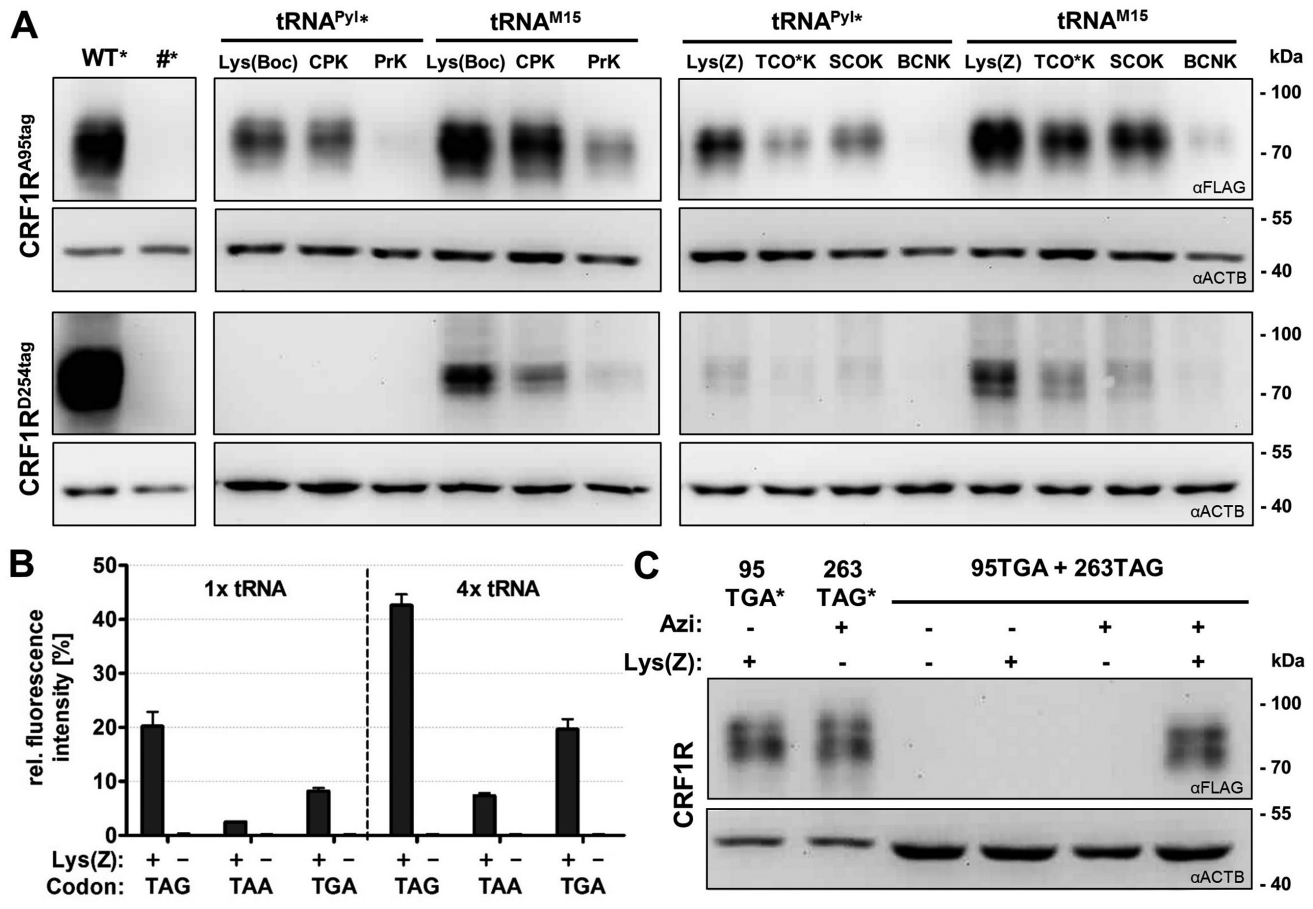
Single and multiple ncAA incorporation into a GPCR. ncAAs were incorporated into the CRF1R bearing FLAG at the C-terminus. (**A**) Amber suppression by the indicated tRNAs combined to either *Mb*PylRS^F^ or *Mb*PylRS^AF^. # indicates non-transfected cells. (**B**) Amber (TAG), opal (TGA) or ochre (TAA) suppression by tRNA^M15^_CUA_, tRNA^M15^_UCA_, tRNA^M15^_UUA_, respectively, measured by fluorescence assay as described above. Data represent the average ± SD of biological triplicates. (**C**) Opal and amber suppression by the *Mb*PylRS^AF^/tRNA^M15^_UCA_ pair combined with the *Ec*AziRS/tRNA^Tyr^_CUA_ pair. In A and C, * indicates half amount and one fourth amount loaded, respectively.

The *Mb*PylRS^AF^/tRNA^M15^ pair was applied to incorporate a series of ncAAs into a demanding protein target, the class B GPCR corticotropin releasing factor type 1 receptor (CRF1R) (Figure 4A). We focused on ncAAs designed for bioorthogonal chemistry, which are a critical application of the Pyl system nowadays. We tested amber suppression both at position 95 in the N-terminal domain (NTD) and position 254 further downstream in extracellular loop 2 (ECL2), which was particularly challenging in previous experiments (18,43). At both positions, tRNA^M15^ yielded higher incorporation of all ncAAs tested in respect to tRNA^Pyl^*, yielding detectable protein amounts in Western blot also when no signal was detectable using tRNA^Pyl^*.

As the PylRS does not recognize the anticodon of its tRNA, pyrrolysine tRNAs can be reassigned to ochre, opal and even four-base codons (44–47). Anticodon variants of tRNA^M15^ functioned as efficient ochre and opal suppressors, with the opal variant giving higher yield than the ochre variant (Figure 4B). Different from what reported for *E. coli* (48), we did not observe appreciable near-cognate read through of any of the stop codons. By combining the *Mb*PylRS^AF^/tRNA^M15^_UCA_ pair with the amber-suppressing *Ec*AziRS/tRNA^Tyr^_CUA_ pair, we simultaneously incorporated two different ncAAs into the CRF1R. Based on densitometric analysis of Western blot signals, the yield of double stop codon suppression was about 25% respect to the single suppression (Figure 4C).

**Figure 5.**
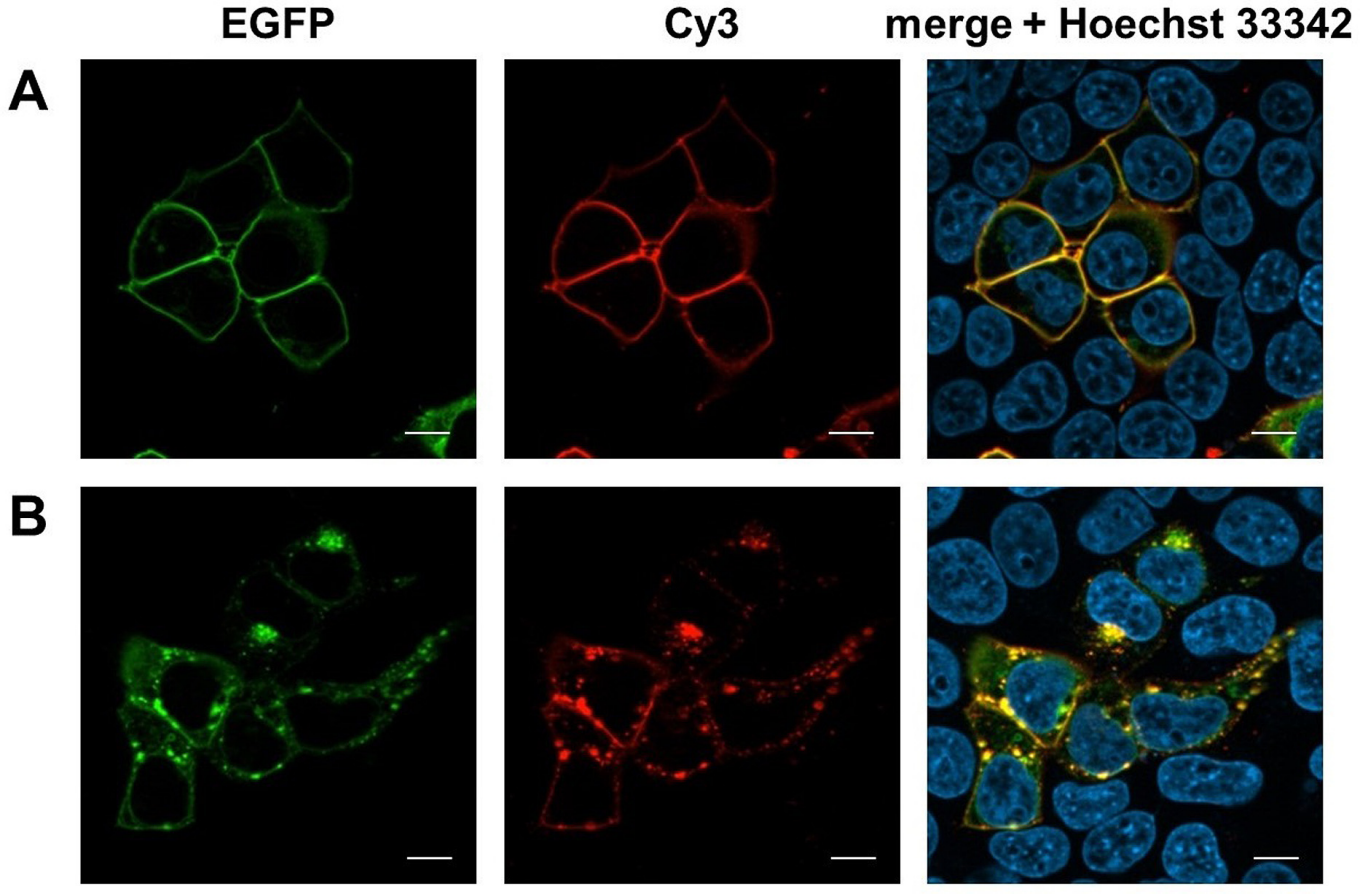
GPCR labeling via SPIEDAC on live cells. Representative images in the green, red and blue channel. (**A**) Cells expressing CRF1R^95TCO*K^-EGFP were treated with Cy3-tetrazine (1.5 μM) for 5 min. Only cells expressing the receptor (green) showed Cy3 labeling (red). (**B**) Cy3-CRF1R^95TCO*K^-EGFP was activated with Urocortin1 (200 nM) and imaged after 15 min. Intracellular vesicles correspond to the internalized receptor. Scale bar: 10 μm.

We also performed labeling and imaging experiments on CRF1R bearing TCO*K (Figure 2A) at position 95^NTD^. The receptor was fused to EGFP to evaluate its expression and membrane localization. Both the number of fluorescent cells and the intensity of EGFP fluorescence increased when using tRNA^M15^ instead of tRNA^Pyl^* (Supplementary Figure S7). CRF1R^95TCO*K^ was selectively labeled via strain promoted inverse-electron-demand Diels-Alder cycloaddition (SPIEDAC) (38) upon a short treatment with Cy3-tetrazine, with the fluorescence of the label localizing with EGFP fluorescence (Figure 5A). The Cy3-labeled CRF1R was still functional, and internalized when challenged with the natural agonist Urocortin1 (Figure 5B).

### The optimal pyrrolysine system for ncAA incorporation in mammalian cells

Recently, Nikic *et al.* discovered that the PylRS accumulates in the nucleus of mammalian cells, so that the suppression efficiency of the Pyl system is enhanced by adding to the PylRS a strong N-terminal nuclear export signal (NES) (49). We combined tRNA^Pyl^*, tRNA^M15^ and tRNA^C15^ with the PylRS bearing the published NES and evaluated the suppression efficiency via fluorescence assay (Supplementary Figure S8). At optimal conditions for ncAA incorporation (0.5 mM ncAA, four copies of tRNA expression cassette), adding the NES was beneficial in the case of tRNA^Pyl^*, but did not improve amber suppression by either tRNA^M15^ or tRNA^C15^. However, when working either with limiting ncAA concentration (50 μM) or a single copy of tRNA expression cassette, a general improvement via the NES was observed. Therefore, the combination of the NES-PylRS with the tRNA^M15^ should guarantee the best performance of the Pyl system for demanding applications.

## DISCUSSION

We reported here the generation of two enhanced orthogonal tRNAs, tRNA^M15^ and tRNA^C15^, for the Pyl orthogonal system, which is the most popular tool for ncAA incorporation into proteins expressed in mammalian cells. The engineered tRNAs feature a 2–5-fold higher intracellular concentration compared to tRNA^Pyl^*, although all tRNA genes are expressed using the same external promoter in identical expression cassettes. Increasing tRNA amounts is known to increase suppression efficiency in mammalian hosts (13,43,50). Our results demonstrate (Figure 3) that higher tRNA concentrations can be better achieved by improving the quality of the tRNA, rather than by increasing the number of tRNA expression cassettes in the expression system, as it has been done so far (13,33).

Comparison of electrophoretic mobility suggests that the engineered tRNAs are intrinsically more stable than the tRNA^Pyl^*. This is possibly due to stronger tertiary interactions, as both tRNA^M15^ and tRNA^C15^ have the tertiary Watson-Crick pair U19:A56 mutated to the more stable pair G19:C56 between D- and T-loop, as it occurs in canonical tRNAs. Literature data (13,14) and the results on *Dh*tRNA^Pyl^ (Supplementary Figure S2) clearly show that tRNA efficiency increases by GC stabilizing tRNA arms. Our engineered tRNAs suggest that the presence of a canonical hinge is likewise important for tRNA efficiency. Interestingly, some recently discovered non-methanosarcinal tRNA^Pyl^ species may feature a canonical hinge (51,52).

Higher structural stability is likely one factor contributing to the higher intracellular levels of engineered tRNAs respect to tRNA^Pyl^*. For instance, it is known that tRNA concentrations are strongly affected by the stability surveillance of the CCA-adding enzyme (53). However, steady state tRNA levels are regulated by complex tRNA turnover mechanisms (54). It is possible that the engineered tRNAs interact with endogenous proteins differently from the tRNA^Pyl^, which may have a protective effect, but elucidating this question will require further investigations.

Interaction of tRNA^Pyl^ variants with the mammalian elongation factor may involve the junction between the acceptor stem and the T-arm. tRNAs mutated in this region (Mutants 18–21) show higher suppression efficiency when charged with Lys(Boc) rather than with Lys(Z). These mutants, like all the chimeras, bear a G7:C66 pair at the root of the acceptor stem, whereas mutants 1–17, which show parallel trends with the two ncAAs, feature the inverted combination C7:G66. Mutations in the same tRNA region have been shown to improve interaction of archaeal suppressor tRNAs with the bacterial elongation factor EF-Tu (15,16). It is also well known that the affinity of AA-tRNAs for EF-Tu depends on the esterified amino acids (55,56). Overall, our results support the notion that suppressor tRNAs should be optimized for each ncAA (15,16).

Orthogonal tRNAs used in mammalian cells are usually derived from prokaryotes to avoid cross talk with the endogenous translational pairs. Here, our tRNA^C15^ demonstrates that an engineered mitochondrial tRNA can well function as orthogonal suppressor tRNAs in the cell cytosol, in line with the prokaryotic origin of mitochondria (30,31). Notably, tRNA^C15^ yielded the highest incorporation efficiency of ncAAs accepted by the PylRS^F^ when using one copy of the tRNA cassette, which may be advantageous when working with viral vectors that do not tolerate the presence of repeated sequences.

ncAAs for ultrafast bioorthogonal chemistry encoded by the PylRS (1) (TCO*K, SCOK, BCNK, Figure 1A) offer the unique possibility of installing biophysical probes, such as last-generation fluorophores for single molecule microscopy, at specific protein sites directly in the live cell. Using our tRNA^M15^, we have demonstrated for the first time ncAA incorporation by the Pyl system into a GPCR, a notoriously more challenging object than model systems like the green fluorescent protein and cytosolic proteins. We achieved rapid fluorescent GPCR labeling via SPIEDAC chemistry on the live cell and we could follow in real time the physiological intracellular fate of the labeled receptor upon stimulation. Our work opens the way to a number of in cell fluorescence experiments with GPCRs, whereas so far site-specific GPCR labeling with small fluorophores had been achieved only on isolated receptors *in vitro* (57). Moreover, we could incorporate two distinct ncAAs into the receptor, thus moving a crucial step toward the development of small intramolecular FRET sensors for *in cell* studies.

Finally, by pairing our engineered tRNA^M15^ with the NES-PylRS, we obtained the most robust version of the Pyl system available to date to incorporate ncAAs even under sub-optimal conditions. We anticipate that this system will find general application for ncAA incorporation into biologically relevant substrates in mammalian cells and facilitate employing ncAA tools to study molecular mechanisms of biological processes in the cellular context.

## Supplementary Material

Supplementary Data
