# Supplementary material for: Designer tRNAs for efficient incorporation of non-canonical amino acids by the pyrrolysine system in mammalian cells
Source: Nucleic Acids Res. 2017 Nov 21;46(1):1–10. doi: 10.1093/nar/gkx1156 (PMC5758916; doi:10.1093/nar/gkx1156)
Supplement: Supplementary Data [file gkx1156_supp.pdf]

Designer tRNAs for efficient incorporation of non-canonical amino acids by the pyrrolysine system in mammalian cells

Robert Serfling, Christian Lorenz, Maja Etzel, Gerda Schicht, Thore Böttke, Mario Mörl, Irene Coin*

Institute of Biochemistry, Faculty of Life Sciences, University of Leipzig, Brüderstraße 34, 04103 Leipzig, Germany

* To whom correspondence should be addressed. Tel: +49 (0)341 9736980; Fax: +49 (0)341 9736909; Email: irene.coin@uni-leipzig.de

Supplementary data

Table S1. Coding-strand sequences of all tRNAs used in this study. Sequences of reference tRNAs are written in bold. Nucleotides that differ from reference are marked either in green for *M.mazei* and *D. hafniense* $tRNA^{Pyl}$ variants or in yellow for *B. taurus* $mt\text{-}tRNA^{Ser}$ chimeric tRNAs. ▼Chimera 1 and Chimera 2 have been described in the literature (1).

	3′ acceptor stem		D-arm		anticodon-arm		T-arm	5′ acceptor stem
	1	8	10 15 20 25		27 29ᵃ30 40 41ᵇ	45	49 55 60 65	66 70 73
***Mm*tRNA^Pyl^ wt**	**GGAAACC**	**T**	**GATCATGTAGATC**	**G**	**AATGGACTCTAAATCCGTT**	**CAG**	**CCGGGTTAGATTCCCGG**	**GGTTTCCG**
$tRNA^{Pyl*}$	GGAAACC	T	GATCATGTAGATC	G	AACGGACTCTAAATCCGTT	CAG	CCGGGTTCGATTCCCGG	GGTTTCCG
$tRNA^{Pyl°}$	GGAAACC	T	GATCATGTAGATC	G	AATGGACTCTAAATCCATT	CAG	CCGGGTTAGATTCCCGG	GGTTTCCG
Mutant 1	GGAAACC	T	GATCATGTAGATC	G	AATGGACTCTAAATCCGTT	CAG	CCGGGTTCGATTCCCGG	GGTTTCCG
Mutant 2	GGAAACC	T	GATCATGTAGATC	G	AATGGACTCTAAATCCGTT	CAG	CCGGGTTAAATTCCCGG	GGTTTCCG
Mutant 3	GGAAACC	T	GATCATGTAGATC	G	AATGGATTCTAAATCCGTT	CAG	CCGGGTTAGATTCCCGG	GGTTTCCG
Mutant 4	GGAAACC	T	GATCATGTAGATC	G	AATGGACTCTAAATCCGTT	CAA	CCGGGTTAGATTCCCGG	GGTTTCCG
Mutant 5	GGAAACC	T	GATCAAGTAGATC	G	AATGGACTCTAAATCCGTT	CAG	CCGGGTTAGATTCCCGG	GGTTTCCG
Mutant 6	GGAAACC	T	GATCAGGTAGATC	G	AATGGACTCTAAATCCGTT	CAG	CCGGGTTAGATTCCCGG	GGTTTCCG
Mutant 7	GGAAACC	T	GTTCATGTAGAAC	G	AATGGACTCTAAATCCGTT	CAG	CCGGGTTAGATTCCCGG	GGTTTCCG
Mutant 8	GGAAACC	T	GGTCATGTAGACC	G	AATGGACTCTAAATCCGTT	CAG	CCGGGTTAGATTCCCGG	GGTTTCCG
Mutant 9	GGAAACC	T	GCTCATGTAGAGC	G	AATGGACTCTAAATCCGTT	CAG	CCGGGTTAGATTCCCGG	GGTTTCCG
Mutant 10	GGAAACC	T	GATCATGGAGATC	G	AATGGACTCTAAATCCGTT	CAG	CCGGGTTAGATTCCCGG	GGTTTCCG
Mutant 11	GGAAACC	T	GATCATGGAGATC	G	AATGGACTCTAAATCCGTT	CAG	CCGGGTTCGATTCCCGG	GGTTTCCG
Mutant 12	GGAAACC	T	GATCATGGAGATC	G	AACGGACTCTAAATCCGTT	CAG	CCGGGTTCGATTCCCGG	GGTTTCCG
Mutant 13	GGAAACC	T	GGTCATGGAGACC	G	AACGGACTCTAAATCCGTT	CAG	CCGGGTTCGATTCCCGG	GGTTTCCG
Mutant 14	GGAAACC	T	GGTCAAGGAGACC	G	AACGGACTCTAAATCCGTT	CAG	CCGGGTTCGATTCCCGG	GGTTTCCG
Mutant 15	GGAAACC	T	GGTCAGGGAGACC	G	AACGGACTCTAAATCCGTT	CAG	CCGGGTTCGATTCCCGG	GGTTTCCG
Mutant 16	GGAAACC	T	GGCCAAGGAGGCC	G	AACGGACTCTAAATCCGTT	CAG	CCGGGTTCGATTCCCGG	GGTTTCCG
Mutant 17	GGAAACC	T	GGCCAGGGAGGCC	G	AACGGACTCTAAATCCGTT	CAG	CCGGGTTCGATTCCCGG	GGTTTCCG
Mutant 18	GGAAACG	T	GGTCAGGGAGACC	G	AACGGACTCTAAATCCGTT	CAG	CCGGGTTCGATTCCCGG	CGTTTCCG
Mutant 19	GGAAACG	T	GGTCAGGGAGACC	G	AACGGACTCTAAATCCGTT	CAG	CGGGGTTCGATTCCCCG	CGTTTCCG
Mutant 20	GGAAACG	T	GGTCAGGGAGACC	G	AACGGACTCTAAATCCGTT	CAG	TGGGGTTCGATTCCCCA	CGTTTCCG
Mutant 21	GGAAACG	T	GGCCAGGGAGGCC	G	AACGGACTCTAAATCCGTT	CAG	TGGGGTTCGATTCCCCA	CGTTTCCG
***Bt*-mttRNA$^{Ser}_{CUA}$**	**GAGAGAG**	**A**	**CATAGAGGTTATG**	**A**	**TGTTGGCTCTAAACCAACA**	**GTA**	**GAAGGTTCGATTCCTTC**	**CTCTCTTA**
Chimera 1▼	GAGAGAG	T	GATCGAGGTGATC	G	TGTTGACTCTAAATCAACA	CAG	GAGGGTTCGATTCCCTC	CTCTCTCG
Chimera 2▼	GAGAGAG	T	GATCGAGGTGATC	A	TGTTGACTCTAAATCAACA	GAG	GAGGGTTCGATTCCCTC	CTCTCTCG
Chimera 3	GAGAGAG	A	GATCGAGGTGATC	G	TGTTGACTCTAAATCAACA	CAG	GAGGGTTCGATTCCCTC	CTCTCTCG
Chimera 4	GAGAGAG	T	GATCGAGGTGATC	A	TGTTGACTCTAAATCAACA	CAG	GAGGGTTCGATTCCCTC	CTCTCTCG
Chimera 5	GAGAGAG	A	GATCGAGGTGATC	A	TGTTGACTCTAAATCAACA	CAG	GAGGGTTCGATTCCCTC	CTCTCTCG
Chimera 6	GAGAGAG	T	GATCAAGGTGATC	G	TGTTGACTCTAAATCAACA	CAG	GAGGGTTCGATTCCCTC	CTCTCTCG
Chimera 7	GGGAGAG	T	GATCGAGGTGATC	G	TGTTGACTCTAAATCAACA	CAG	GAGGGTTCGATTCCCTC	CTCTCCCG
Chimera 8	GGGAGAG	T	CATAAAGGTTATG	A	TGTTGGCTCTAAACCAACA	GTA	GGGGGTTCGATTCCCCC	CTCTCCCG
Chimera 9	GGGAGAG	T	GATAAAGGTTATC	A	TGTTGGCTCTAAACCAACA	GTA	GGGGGTTCGATTCCCCC	CTCTCCCG
Chimera 10	GGGAGAG	T	GATAAAGGTTATC	G	TGTTGGCTCTAAACCAACA	CAG	GGGGGTTCGATTCCCCC	CTCTCCCG
Chimera 11	GGGAGAG	T	GATCAAGGTGATC	G	TGTTGACTCTAAATCAACA	CAG	GGGGGTTCGATTCCCCC	CTCTCCCG
Chimera 12	GGGAGAG	T	GATCAAGGTGATC	G	TGTTGACTCTAAATCAACA	CAG	GAGGGTTCGATTCCCTC	CTCTCCCG
Chimera 13	GGGAGAG	T	GGTCAAGGTGACC	G	TGTTGACTCTAAATCAACA	CAG	GGGGGTTCGATTCCCCC	CTCTCCCG
Chimera 14	GGGAGAG	T	GACCAAGGTGGTC	G	TGTTGACTCTAAATCAACA	CAG	GGGGGTTCGATTCCCCC	CTCTCCCG
Chimera 15	GGGAGAG	T	GGCCAAGGTGGCC	G	TGTTGACTCTAAATCAACA	CAG	GGGGGTTCGATTCCCCC	CTCTCCCG
***Dh*tRNA^Pyl^ wt**	**GGGGGGT**	**G**	**GATCGAATAGATC**	**A**	**CACGGACTCTAAATTCGTG**	**CAG**	**GCGGGTGAAACTCCCGT**	**ACTCCCCG**
Dh$tRNA^{Pyl*}$	GGGGGGT	G	GATCGAATAGATC	A	CACGGACTCTAAATCCGTG	CAG	GCGGGTGAAACTCCCGC	ACCCCCCG

Table S2: Suppression efficiency and orthogonality of tRNA variants paired with *Mb*PylRS^F, determined via the fluorescence assay in the presence and absence of Lys(Boc). Orthogonal tRNAs are not recognized by endogenous synthetases and should give no amber suppression (*i.e.* no fluorescence) when no ncAA is present.

tRNA (1x copy)	Relative fluorescence intensity in % [to wild type EGFP]					
	+ ncAA	SD	n	- ncAA	SD	n
non transfected HEK293	0.06	0.04	4	0.07	0.00	2
tRNA^{Pyl*}	9.04	0.77	5	0.06	0.00	2
M1	3.97	0.48	3	0.07	0.01	2
M2	3.73	0.41	3	0.06	0.01	2
M3	1.87	0.32	3	0.03	0.04	2
M4	0.90	0.32	3	0.02	0.04	2
M5	9.10	1.16	3	0.04	0.02	2
M6	11.20	0.93	3	0.03	0.03	2
M7	6.73	0.52	3	0.04	0.03	2
M8	12.97	1.08	3	0.04	0.02	2
M9	12.50	0.67	3	0.05	0.03	2
M10	2.13	1.08	3	0.05	0.02	2
M11	15.10	2.13	4	0.04	0.02	2
M12▼	17.10	2.62	4	0.05	0.01	2
M13▼	18.76	2.88	4	0.06	0.01	2
M14▼	15.52	1.66	4	0.06	0.01	2
M15▼	19.26	1.53	4	0.03	0.04	2
M16▼	13.58	2.05	3	0.32	0.01	2
M17▼	16.28	2.37	3	0.23	0.00	2
M18▼	15.82	1.70	4	0.21	0.03	2
M19▼	19.44	2.13	4	0.15	0.00	2
M20▼	21.50	2.29	4	0.09	0.00	2
M21▼	17.18	2.77	3	0.31	0.05	2
C1	10.60	2.35	3	0.05	0.01	2
C2	1.50	1.10	3	0.04	0.02	2
C3	0.73	0.38	3	0.04	0.02	2
C4	7.07	2.27	3	0.04	0.02	2
C5	4.50	2.76	3	0.06	0.03	2
C6	12.47	2.03	3	0.07	0.03	2
C7	11.73	3.11	3	0.04	0.02	2
C8	0.40	0.20	3	0.04	0.02	2
C9	0.67	0.33	3	0.04	0.02	2
C10	2.57	0.32	3	0.03	0.02	2
C11	25.35	4.15	2	0.03	0.02	2
C12▼	24.83	3.22	3	0.04	0.02	2
C13▼	18.16	2.41	4	0.03	0.03	2
C14▼	20.69	3.08	4	0.04	0.03	2
C15▼	30.57	2.74	4	0.04	0.02	2

▼data acquired using the codon optimized *Mb*PylRS^F.

Table S3. Aminoacylation of tRNA^{Pyl*}, tRNA^{M15} and tRNA^{C15} with Lys(Boc) and Lys(Z). Data were obtained from densitometric analysis of Northern blots and represent the percentage of AA-tRNA compared to total tRNA present in the sample, as follows:

$$Aminoacylation\ rate\ in\ \% = \frac{amount\ of\ AA\text{-}tRNA}{amount\ of\ AA\text{-}tRNA + amount\ of\ tRNA} * 100\%$$

	Lys(Boc)			Lys(Z)		
	**Aminoacylation rate**	**± SD**	**n**	**Aminoacylation rate**	**± SD**	**n**
tRNA^{Pyl*} (1x)	50.1	5.8	3	49.8	3.4	3
tRNA^{Pyl*} (4x)	44.4	1.9	3	47.2	1.5	3
M15 (1x)	45.8	4.5	3	46.7	3.1	3
M15 (4x)	44.8	6.4	3	45.8	4.9	3
C15 (1x)	46.3	5.3	3	50.6	6.7	3
C15 (4x)	47.7	4.3	3	51.6	5.0	3

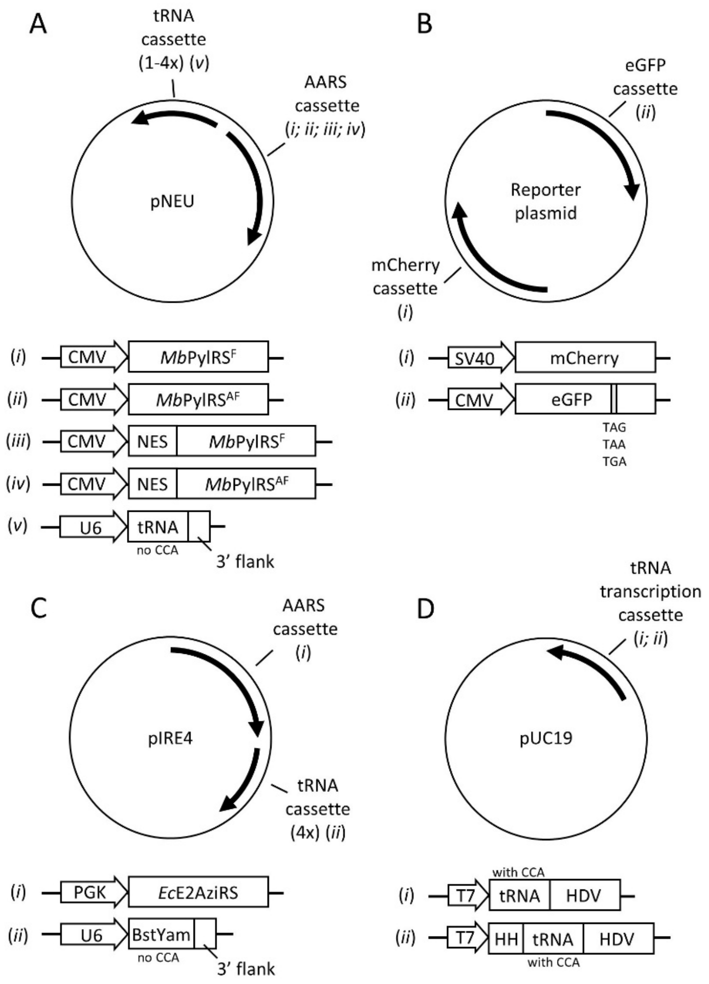

Figure S1. Plasmids used in this study. (**A**) pNEU. The backbone of pNEU is essentially the same as pcDNA3.1 with some variations in the restriction sites. Most important, *Sal*I and *Xba*I restriction sites were introduced upstream the CMV promoter and removed from other regions of the vector. (**B**) Reporter plasmid for the fluorescence assay. EGFP was cloned into the multi cloning site of pcDNA3.0 between *EcoR*I and *Xho*I sites. Amber (TAG), opal (TGA) and ochre (TAA) stop-codon were introduced at the permissive site Y183. mCherry replaces the original neomycin resistance gene of pcDNA3.0 between *Xma*I and *BstB*I sites. (**C**) pIRE4. The backbone of pIRE4 is based on pEGFP-N1 (Clontech, Mountain View, CA), with major modifications. The CMV-EGFP sequence of pEGFP-N1 was substituted with the AARS cassette, followed by four copies of the tRNA cassette right downstream the polyadenylation sequence, similar to pShax (2). The tRNA gene is under control of the U6 promoter (3). (**D**) pUC19. Cassettes for *in vitro* tRNA transcription were cloned into a pUC19 vector between *EcoR*I and *Pst*I restriction sites. To produce tRNA standards for Northern blot analysis, the tRNA gene was 3'-flanked by a self-cleaving hepatitis delta virus (HDV) ribozyme to guarantee homogeneity of the 3'-end (4). To produce tRNA samples for structural probing, an additional hammerhead (HH) ribozyme was added at the 5'-site, which obviates the need for dephosphorylating the 5'-end prior to radiolabeling (4). HDV-cleavage leaves a 2',3'-cyclic phosphate product at the 3', so that tRNAs need to be 3'-dephosphorylated. Both constructs were driven by a T7 promoter.

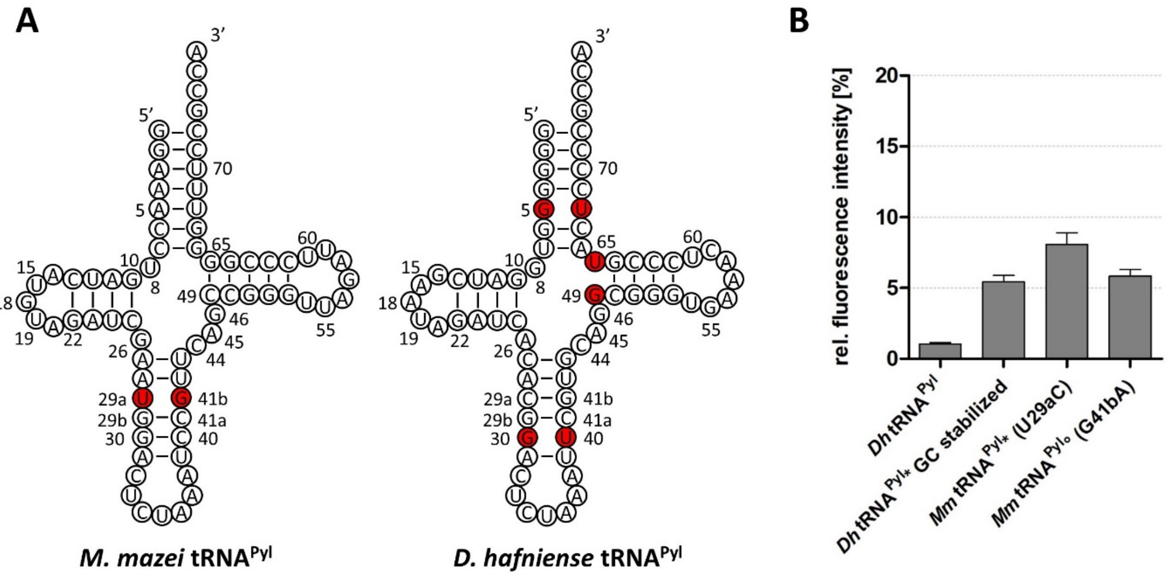

Figure S2: Comparing amber-suppression efficiency of tRNA^{Pyl} from *M. mazei* and *D. hafniense*. (**A**) Cloverleaf structures of wild type *Mm*tRNA^{Pyl} and *Dh*tRNA^{Pyl}. Nucleotides that do not form Watson-Crick base-pairs in the wild tape tRNAs are highlighted in red. (**B**) Amber codon suppression in EGFP^{183TAG} by different tRNA^{Pyl} variants (1x copy) combined with MbPylRS^{F} for incorporation of Lys(Boc). Data represent the average ± SD of biological triplicates. *Dh*tRNA^{Pyl*} indicates the GC stabilized variant of the *Dh*tRNA^{Pyl} (U40C, U65C, U68C). Note that the U29aC mutation is more efficient that the G41bA mutation to stabilize the *Mm*tRNA^{Pyl}.

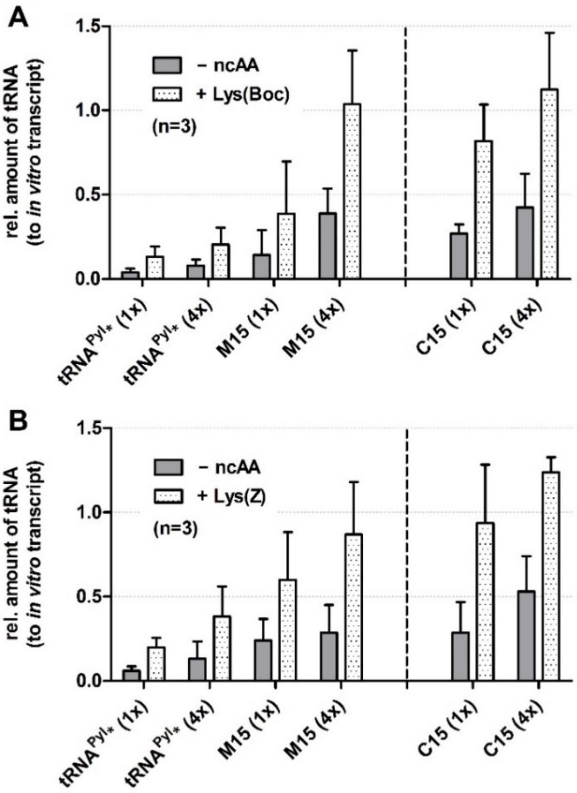

Figure S3: Densitometry analysis of tRNA amounts on Northern blots. Bars represent intensity of bands relative to the corresponding *in vitro* transcripts (average ± SD of biological triplicates). Experiments were performed in the presence and absence of either (**A**) Lys(Boc) or (**B**) Lys(Z). Total tRNA per sample is given as sum of the amount of non-acylated tRNA plus the amount of AA-tRNA.

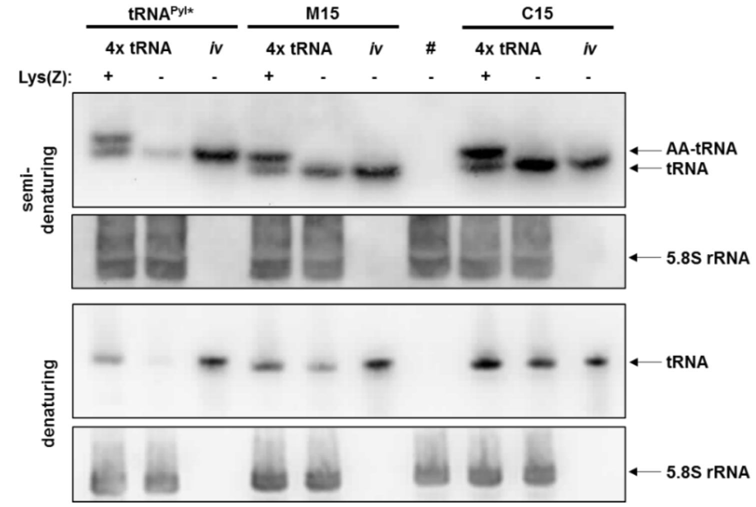

Figure S4. Migration of $tRNA^{Pyl}$ variants in PAGE analysis. Samples were resolved on 10.0% urea-PAA gels under semi-denaturing conditions (top) and denaturing conditions (bottom). Hybridization with biotin-labeled $tRNA^{Pyl*}$/$tRNA^{M15}$ and $tRNA^{C15}$ probes together with 100x excess of unlabeled unfolder-primers. For each *in vitro* (*iv*) tRNA a total of 25 ng was loaded. Ribosomal 5.8S RNA was used as loading control. # indicates RNA probes extracted from non-transfected cells.

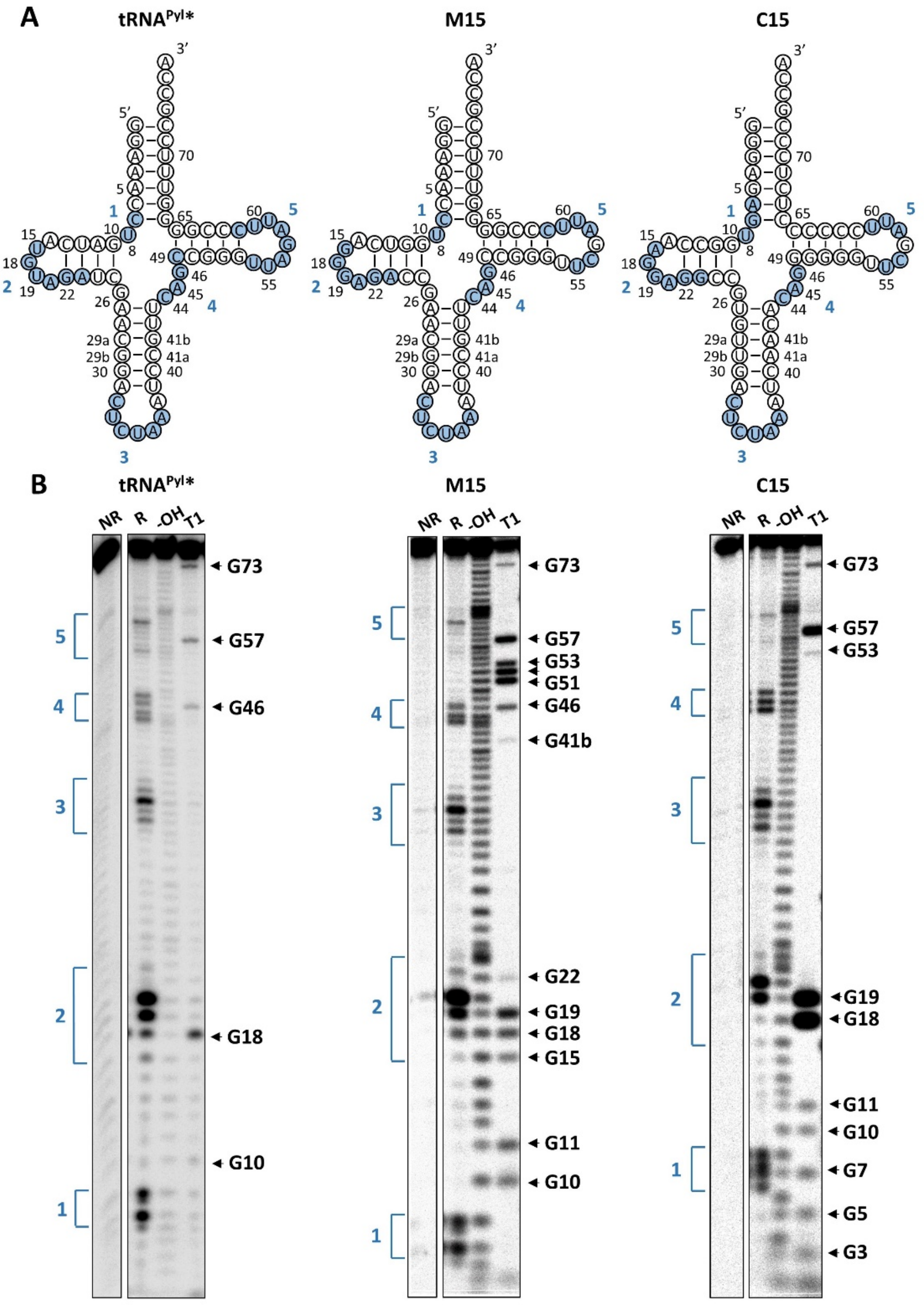

Figure S5: In-line structure probing of different tRNA variants. (**A**) Secondary structure model and (**B**) in-line structure probing of tRNA^{Pyl*}, M15 and C15. RNA differentially degrades according to its secondary structure upon incubation in a mildly alkaline buffer (probing buffer, pH 8.5) (5). When the 2' oxygen of the ribose, the phosphorus, and the adjacent 5' oxygen enter an “in-line” conformation, the 2' oxygen acts as a nucleophile and displaces the 5’ oxygen in the neighboring phosphorus center, which cleaves the RNA linkage. Single-stranded RNA regions are highly susceptible to spontaneous cleavage while structured regions of folded RNA (e.g. base paired helical regions) have a reduced cleavage chance (6). (**B**) Lane 1 (NR, no-reaction): radio-labeled 5’^{32}P-tRNA incubated in water; lane 2 (R, reaction): radio-labeled 5’^{32}P-tRNA incubated with in-line probing buffer for 40 h; lane 3 (-OH): sequencing ladder from alkali treatment; lane 4 (T1): sample digested with RNase T1. Black arrows

indicate guanine positions and clamps indicate cleavage regions within the RNA. Corresponding regions are highlighted in blue in the secondary structure model. All tRNAs gave similar cleavage patterns at the four single stranded loop regions (D-loop, anticodon-loop, variable-loop and TΨC-loop) and at N8-N9 linking the acceptor stem and D-stem. The T-loop of all tRNAs displayed reduced cleavage, most likely due to reduced flexibility of the structurally conserved T-loop motif, which is stabilized by a reverse Hoogsteen base-pair between U54 and A58, a base-stack between a pyrimidine at position U55 and U54, and additional hydrogen bonds with a D-loop guanosine, which in turn is inserted between A58 and G57 forming a three-nucleotide continuous base-stack (7).

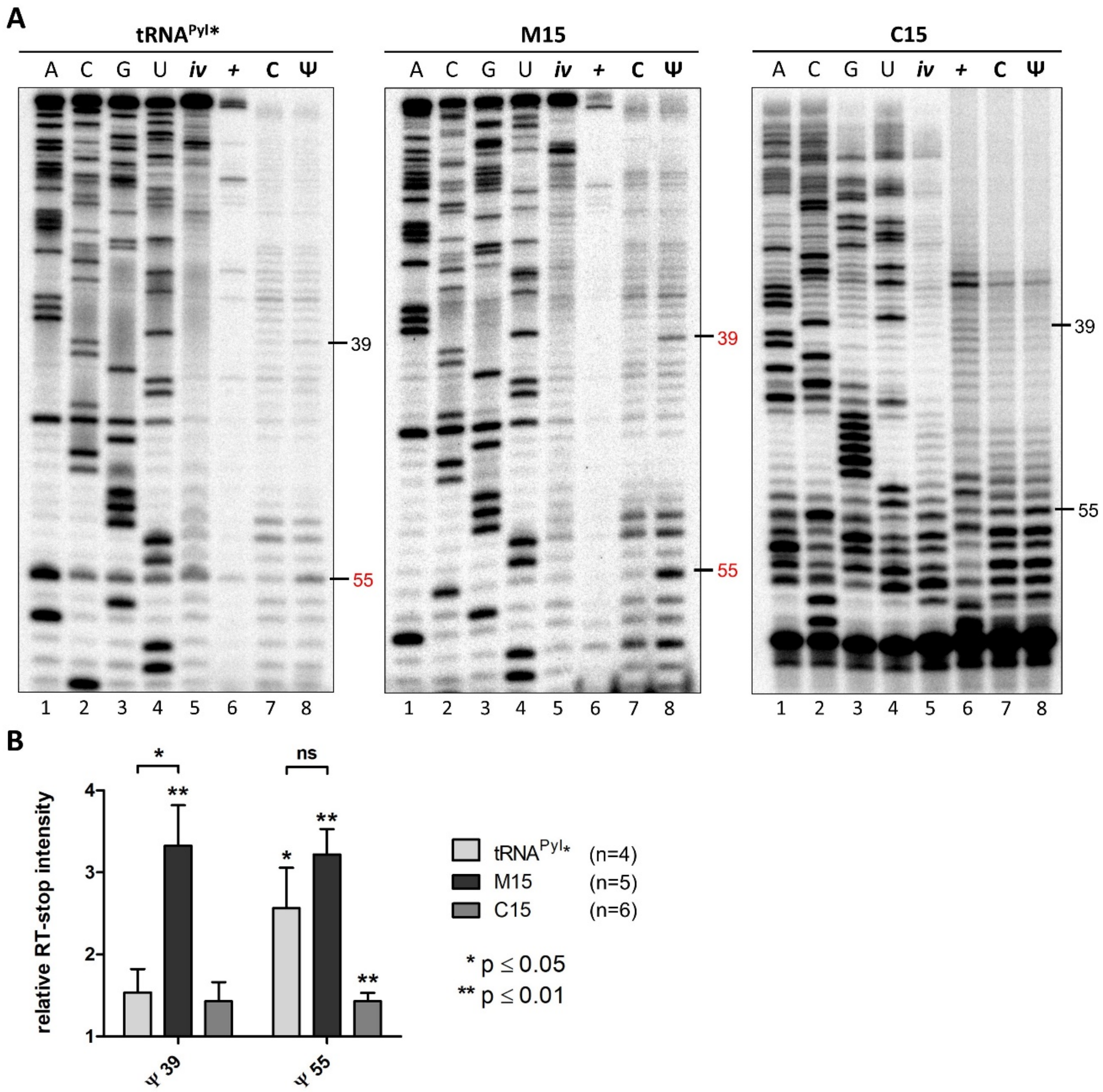

Figure S6: Detection of post-transcriptional modifications. (**A**) Each panel represents reverse transcription (RT) profiles of the indicated tRNA. Extension reactions were performed using ^{32}P-labeled primers and RT-products were resolved on 15% denaturing urea-PAA gels. Lanes 1-4: sequencing ladder indicating A, C, G and U positions; Lane 5: *in vitro* (*iv*) transcript; Lanes 6-8: RNA sample

extracted from HEK293 cells expressing the desired tRNA; Lane 6 (+): no pre-treatment; Lane 7 (C): pre-treatment with Na_2CO_3 solution; Lane 8 (Ψ): pre-treatment with CMCT followed by Na_2CO_3 solution. The method relies on the occurrence of RT-pauses during a primer extension reaction on a tRNA template (8). RT-pauses occur at modified nucleotides due to impaired Watson-Crick pairing with dNTPs during the elongation reaction, which yield cDNA segments truncated one base before the mutated position. To enhance this effect, tRNA samples are treated with selective chemical reagents targeting a given type of modified nucleotide before the RT-reaction. However, RT-pauses can happen also because of unpredictable effects of the RNA primary and secondary structure (8). In general, the RT-profile of any tRNA shows a ladder of DNA products interrupted at different sites. To distinguish RT-pauses caused by the intrinsic structure of the tRNA from pauses caused by post-transcriptional modifications, RT-profiles of tRNAs produced *in vivo* are compared to profiles of the corresponding non-modified *in vitro* transcripts.

To detect pseudouridine (Ψ) via RT, the tRNA is pre-treated with CMCT, which reacts with G, U and Ψ residues and forms bulky G-CMC, U-CMC and Ψ-CMC adducts. Then, G-CMC and U-CMC sites are hydrolyzed back to G and U, respectively, upon extended incubation at alkaline pH, which does not affect the more stable Ψ-CMC adduct. RT of this sample gives truncated products caused both by tRNA hydrolysis during the alkaline treatment and RT-pauses at Ψ-CMC sites. At pseudouridine sites, samples treated with CMCT and Na_2CO_3 (lane 8, Ψ) will give a stronger band in respect to samples treated only with Na_2CO_3 (lane 7, C).

(**B**) Densitometric analysis of radioactive bands generated by RT-pauses. Signals acquired for samples pre-treated with CMCT followed by Na_2CO_3 (lane 8, Ψ) were compared to signals obtained for the control sample treated only with Na_2CO_3 (lane 7, C). The graph illustrates the average ± SD of the ratios between the band intensity of pseudouridine induced RT-stops divided by the band intensity of corresponding RT-stops observed in the alkaline treated control. At least four biological replicates were performed for each tRNA. T-tests were performed to check whether ratios are significantly larger than 1 and to estimate differences between $tRNA^{Pyl*}$ and $tRNA^{M15}$. ns = not significant.

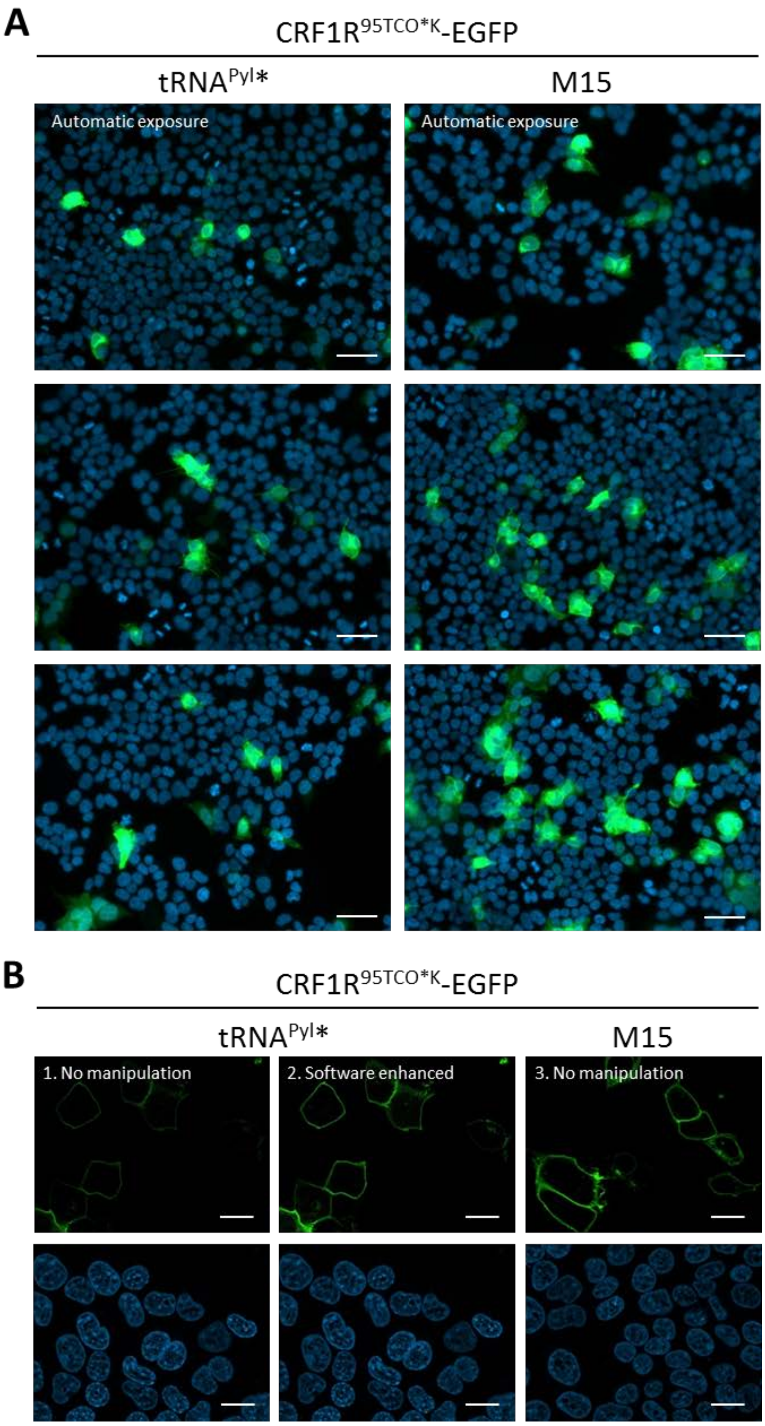

Figure S7: Comparing the expression of full-length CRF1R^{95TCO*K}-EGFP mediated by either tRNA^{Pyl}* or M15. (**A**) Detection of EGFP (green) with a 20x objective, using automatic exposure time. Scale bar: 50 µm. (**B**) Detection of EGFP (green) with a 63x objective. Scale bar: 20 µm. All pictures in the green channel were taken using an identical exposure time of 220 ms. Panel 1 and 2 represent the same picture, but the signal intensity in panel 2 was amplified using Zen 2 lite (Zeiss) software. (**A-B**) Hoechst 33342 staining (blue) of all samples was performed directly prior to microscopy.

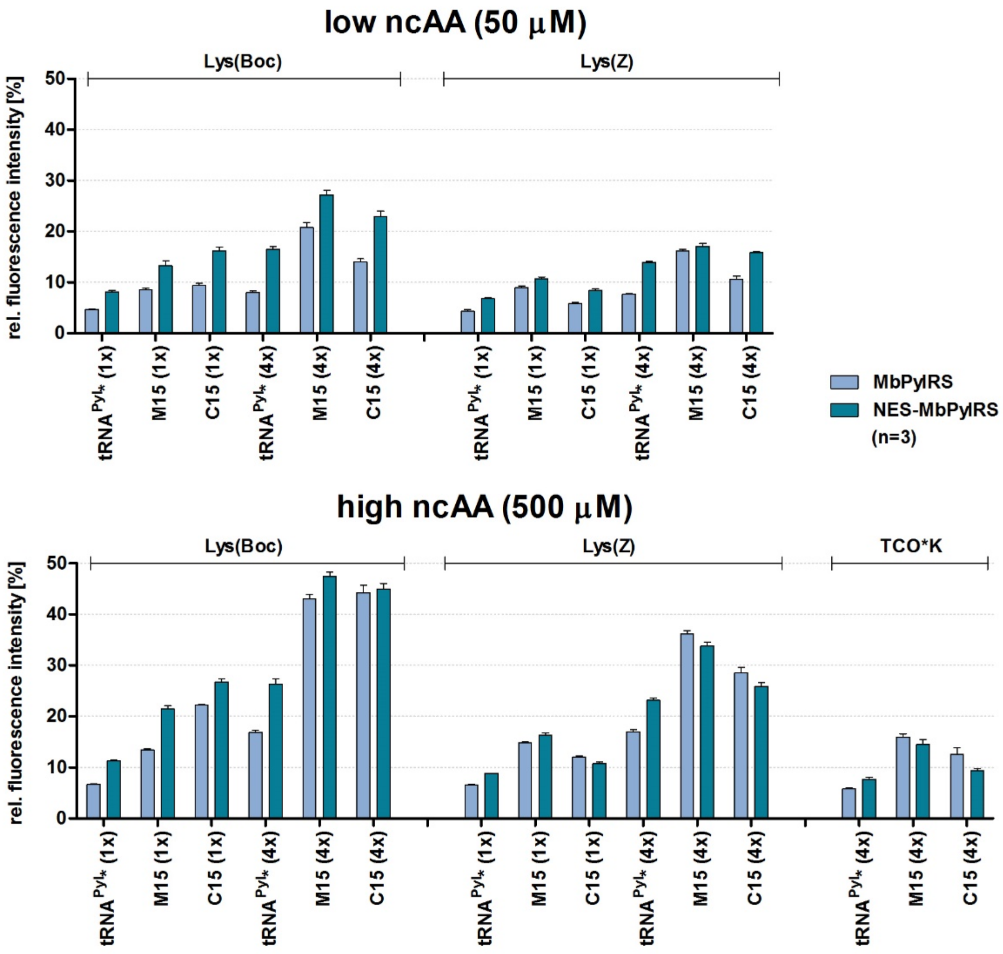

Figure S8: Comparing amber-suppression efficiency of *Mb*PylRS and NES-*Mb*PylRS variants. Incorporation of Lys(Boc) is mediated by either *Mb*PylRS^{F} or NES-*Mb*PylRS^{F} paired with one or four copies of tRNA expression cassettes, while charging tRNA^{Pyl} with Lys(Z) and TCO*K is performed by *Mb*PylRS^{AF} or NES-*Mb*PylRS^{AF}. Data represent the average ± SD of biological triplicates.

Sequences of humanized genes

_Mb_PylRS^{F}, codon optimized for human cells
ATGGACAAGAAACCCCTGGACGTGCTGATCTCCGCCACCGGCCTGTGGATGAGCAGAA
CCGGCACCCTGCACAAGATCAAGCACCACGAGGTGTCCAGAAGCAAGATCTACATCGA
GATGGCCTGCGGCGACCACCTGGTCGTGAACAACAGCAGAAGCTGCCGGACCGCCAG
AGCCTTCCGGCACCACAAGTACAGAAAGACCTGCAAGCGGTGCCGGGTGTCCGACGA
GGATATCAACAACTTCCTGACCAGAAGCACCGAGAGCAAGAACAGCGTGAAAGTGCGG
GTGGTGTCCGCCCCCAAAGTGAAGAAAGCCATGCCCAAGAGCGTGTCCAGAGCCCCCA
AGCCCCTGGAAAATTCCGTGTCCGCCAAGGCCAGCACCAACACCAGCAGATCCGTGCC
CAGCCCTGCCAAGAGCACCCCCAATAGCTCTGTGCCTGCCTCTGCCCCTGCCCCCAGC
CTGACAAGATCCCAGCTGGACAGAGTGGAAGCCCTGCTGAGCCCCGAGGACAAGATCA
GCCTGAACATGGCCAAGCCCTTCAGAGAGCTGGAACCCGAGCTCGTGACCCGGCGGA
AGAACGACTTCCAGCGGCTGTACACCAACGACAGAGAGGACTACCTGGGCAAGCTGGA
ACGGGACATCACCAAGTTCTTCGTGGACCGGGGCTTCCTGGAAATCAAGAGCCCCATC
CTGATCCCCGCCGAGTACGTGGAACGGATGGGCATCAACAACGACACCGAGCTGAGCA
AGCAGATCTTCCGGGTGGACAAAAACCTGTGCCTGAGGCCCATGCTGGCCCCCACCCT
GTACAACTACCTGCGGAAACTGGACCGGATCCTGCCTGGCCCCATCAAGATCTTCGAA
GTGGGCCCCTGCTACCGGAAAGAGAGCGACGGCAAAGAGCACCTGGAAGAGTTTACAA
TGGTCAACTTCTGCCAGATGGGCAGCGGCTGCACCAGAGAGAATCTGGAAGCTCTGAT
CAAAGAGTTCCTGGACTATCTGGAAATCGACTTCGAGATCGTGGGCGACAGCTGCATG
GTGTTCGGCGACACCCTGGACATCATGCACGGCGACCTGGAACTGAGCAGCGCTGTCG
TGGGACCCGTGTCCCTGGATAGAGAGTGGGGCATCGACAAGCCCTGGATCGGAGCCG
GCTTCGGCCTGGAAAGACTGCTGAAAGTGATGCACGGCTTCAAGAACATCAAGCGGGC
CTCCCGCAGCGAGAGCTACTACAACGGCATCTCCACCAACCTGTGA

Codon optimized _Mb_PylRS^{F} added of NES:
The sequence of the nuclear export signal (lowercase letters) was appended to the N-terminal of codon optimized *Mb*PylRS (excerpt written in capital letters):
atggcctgccccgtgcccctgcagctgccacccctggagcgcctgaccctggacATGGACAAGAAACCCCTGGAC
GTGCTGATC…

tRNA expression cassette (Example: tRNA^{Pyl}*):
Restriction sites are underlined. The U6 promoter sequence is written in capital letters. The tRNA^{Pyl}* is written in lowercase letters and the 3'flanking sequence is highlighted in italic.
TCTAGACTCGAGGGGCAGGAAGAGGGCCTATTTCCCATGATTCCTTCATATTTGCATAT
ACGATACAAGGCTGTTAGAGAGATAATTAGAATTAATTTGACTGTAAACACAAAGATATT
AGTACAAAATACGTGACGTAGAAAGTAATAATTTCTTGGGTAGTTTGCAGTTTTAAAATTA
TGTTTTAAAATGGACTATCATATGCTTACCGTAACTTGAAAGTATTTCGATTTCTTGGCTT
TATATATCTTGTGGAAAGGACGAAACACCggaaacctgatcatgtagatcgaacggactctaaatccgttcag
ccgggttagattcccggggtttccg*TTTTTT*GTCGAC

References

1. Ambrogelly, A., Gundllapalli, S., Herring, S., Polycarpo, C., Frauer, C. and Soll, D. (2007) Pyrrolysine is not hardwired for cotranslational insertion at UAG codons. *Proc. Natl. Acad. Sci. U S A*, **104**, 3141-3146.
2. Xiao, H., Chatterjee, A., Choi, S.H., Bajjuri, K.M., Sinha, S.C. and Schultz, P.G. (2013) Genetic incorporation of multiple unnatural amino acids into proteins in mammalian cells. *Angew. Chem. Int. Ed. Engl.*, **52**, 14080-14083.
3. Kunkel, G.R., Maser, R.L., Calvet, J.P. and Pederson, T. (1986) U6 small nuclear RNA is transcribed by RNA polymerase III. *Proc. Natl. Acad. Sci. USA*, **83**, 8575-8579.
4. Mörl, M., Lizano, E., Willkomm, D.K. and Hartmann, R.K. (2008), *Handbook of RNA Biochemistry*. Wiley-VCH Verlag GmbH, pp. 22-35.
5. Regulski, E.E. and Breaker, R.R. (2008) In-line probing analysis of riboswitches. *Methods Mol Biol*, **419**, 53-67.
6. Soukup, G.A. and Breaker, R.R. (1999) Relationship between internucleotide linkage geometry and the stability of RNA. *RNA*, **5**, 1308-1325.
7. Chan, C.W., Chetnani, B. and Mondragon, A. (2013) Structure and function of the T-loop structural motif in noncoding RNAs. *Wiley Interdiscip Rev RNA*, **4**, 507-522.
8. Motorin, Y., Muller, S., Behm-Ansmant, I. and Branlant, C. (2007) Identification of modified residues in RNAs by reverse transcription-based methods. *Methods Enzymol.*, **425**, 21-53.
